# Clinical Application of Shear Wave Elastography for Assisting Brain Tumor Resection

**DOI:** 10.3389/fonc.2021.619286

**Published:** 2021-03-01

**Authors:** Huan Wee Chan, Christopher Uff, Aabir Chakraborty, Neil Dorward, Jeffrey Colin Bamber

**Affiliations:** ^1^ Joint Department of Physics, Institute of Cancer Research and the Royal Marsden Hospital, Sutton, United Kingdom; ^2^ Neurosurgery Department, Southampton General Hospital, Southampton, United Kingdom; ^3^ Neurosurgery Department, Royal London Hospital, London, United Kingdom; ^4^ Neurosurgery Department, The National Hospital for Neurology and Neurosurgery, London, United Kingdom

**Keywords:** brain tumor, detecting brain tumor residual, elastography, shear wave, intraoperative ultrasound

## Abstract

**Background:**

The clinical outcomes for brain tumor resection have been shown to be significantly improved with increased extent of resection. To achieve this, neurosurgeons employ different intra-operative tools to improve the extent of resection of brain tumors, including ultrasound, CT, and MRI. Young’s modulus (YM) of brain tumors have been shown to be different from normal brain but the accuracy of SWE in assisting brain tumor resection has not been reported.

**Aims:**

To determine the accuracy of SWE in detecting brain tumor residual using post-operative MRI scan as “gold standard”.

**Methods:**

Thirty-four patients (aged 1–62 years, M:F = 15:20) with brain tumors were recruited into the study. The intraoperative SWE scans were performed using Aixplorer^®^ (SuperSonic Imagine, France) using a sector transducer (SE12-3) and a linear transducer (SL15-4) with a bandwidth of 3 to 12 MHz and 4 to 15 MHz, respectively, using the SWE mode. The scans were performed prior, during and after brain tumor resection. The presence of residual tumor was determined by the surgeon, ultrasound (US) B-mode and SWE. This was compared with the presence of residual tumor on post-operative MRI scan.

**Results:**

The YM of the brain tumors correlated significantly with surgeons’ findings (*ρ* = 0.845, p < 0.001). The sensitivities of residual tumor detection by the surgeon, US B-mode and SWE were 36%, 73%, and 94%, respectively, while their specificities were 100%, 63%, and 77%, respectively. There was no significant difference between detection of residual tumor by SWE, US B-mode, and MRI. SWE and MRI were significantly better than the surgeon’s detection of residual tumor (p = 0.001 and p < 0.001, respectively).

**Conclusions:**

SWE had a higher sensitivity in detecting residual tumor than the surgeons (94% vs. 36%). However, the surgeons had a higher specificity than SWE (100% vs. 77%). Therefore, using SWE in combination with surgeon’s opinion may optimize the detection of residual tumor, and hence improve the extent of brain tumor resection.

## Introduction

The clinical outcomes for brain tumor resection have been shown to be significantly improved with increased extent of resection (1–18). To achieve this, neurosurgeons employ different intra-operative tools to improve the extent of resection of brain tumors (19–22). Although neuronavigation with pre-operative imaging is indispensable in providing useful information for craniotomy planning, it is susceptible to structural shift during resection (23). Intra-operative MRI (iMRI) has been shown to significantly improve the extent of resection and clinical outcomes (21) of brain tumors by providing high resolution imaging. However, iMRI increases operative time by up to 107 min (21, 24) and is not widely available, especially in the less affluent neurosurgical units. Furthermore, it provides only infrequent (one or two) opportunities to image during surgery. Fluorescence imaging with 5-ALA (5-aminolevulinic acid) has been shown to improve the extent of resection and therefore survival in patients with malignant glioma (22). However, this method is limited to the application in malignant gliomas. The fluorescence is only limited to tumor surface and can be obscured by blood and normal brain tissue (25). By providing real-time intra-operative imaging with nearly unlimited imaging opportunity and minimal effect on operative time, intraoperative US (IOUS) has also been shown to provide significant improvement the extent of resection (26, 27), even without integration with neuronavigation (28). It can also improve quality of life in patients who had brain tumor surgery (29). However, the artifacts of IOUS such as post-resection hyperechoic rim (30, 31), post-surgical and post-radiation artifacts (26), acoustic shadowing from Surgicel (30), peritumoural-oedema hyperechogenicity (32), and hyperechoic blood (32), may pose difficulty in IOUS interpretation. Furthermore, in some cases, IOUS cannot distinguish the surrounding tissue from the tumor (33). Due to artifacts and limitations in IOUS and inaccuracies of neurosurgeons in estimating residual tumor intra-operatively (34, 35), ultrasound elastography may provide differentiation between residual tumor from artifacts on IOUS. Intraoperative contrast-enhanced ultrasound (iCEUS) has been shown to add anatomic and biological information but its utility in detecting tumor remnants have not been studied (36).

For a long time, neurosurgeons rely on visual inspection and tactile feedback to help determine the nature of the tissue being resected during surgery. Ultrasound elastography is an ultrasound-based method of obtaining biomechanical properties of tissue. There are two main types of ultrasound elastography, i.e., qualitative and quantitative elastography. Quasistatic strain elastography (QSE) is a qualitative elastography method whereby the operator applies a certain amount of pressure to deform the tissue (37). The degree to which the tissue deforms is defined as strain. The stiffness will be inversely proportional to the strain. As the amount of pressure applied by the operator, i.e., stress, cannot be accurately quantified, this method can only determine the strain of the tumor in relation to the surrounding brain tissue. On the other hand, shear wave elastography (SWE) is a quantitative elastography technique where stiffness of tissue was obtained by measuring the speed of the shear waves generated in the tissue.

### Shear Wave Elastography

SWE is a type of elasticity imaging technique, which allows quantification of soft tissue elastic modulus. This technique requires generation of shear waves in the tissue either by ARF (38, 39) or mechanically (40–42). Shear waves are secondary waves that propagate perpendicular to the direction of displacement, analogous to circular ripples on the water surface that travels outward when a disturbance is introduced. The shear wave propagation speed is dependent on the Young’s (elastic) modulus of the medium by the equation *E* = 3*ρc* ^2^, where *E* is the Young’s modulus, *ρ* is the medium density, and *c* is the shear wave propagation speed. Assuming that the medium through which the shear waves travel has a density of approximately 1,000 kg/m^3^, the equation becomes *E* ≈ *c* ^2^.

In this study, SWE is performed using SSI (SuperSonic shear imaging) where ARF (acoustic radiation force) is applied to soft tissue to induce displacement to generate perpendicularly propagating shear waves, the speed of which are subsequently estimated with cross-correlation function, thus allowing quantitative real-time mapping of elastic modulus (38). This system is capable of producing an ARF sweep to successively focus on different depths along the line of excitation in a Mach cone, thus allowing generation of shear waves at multiple depths, known as quasi-plane shear waves (43). As a result of the Mach cone, the shear waves generated are shaped like a cone, which is at an angle to the axis of excitation travelling in opposite directions to each other.

Ultrafast imaging acquisition is performed using plane wave transmit-receive. This means that the whole 128 elements are fired at the same time, therefore, for a 3-cm imaging depth, the achievable frame rate is ~25 kHz, more than 100 times that of conventional ultrasound. This means that the ultrafast imaging regime can capture up to three frames of the shear waves travelling within 1 mm, thus allowing real-time elasticity mapping or elastography.

## Methods

### Patient Selection

Patients were recruited prospectively from Great Ormond Street Hospital for Children and The National Hospital for Neurology and Neurosurgery between September 2011 and May 2013. This study was approved by the National Research Ethics Service Committee London – Queen Square. The inclusion criteria were as follows:

1. They were diagnosed with brain tumor.
2. They had consented to undergo craniotomy and resection or open biopsy of the tumor.
3. They have given their consent for this study, or their parents have given their consent on their behalf for this study if they are under 16 years old.

Those who had consented for craniotomy but underwent neuronavigation-guided biopsy (burr hole biopsy), were excluded because the surgeon would be unable to comment on the stiffness of the tumor, and it would not be possible to perform intra-operative SWE with the SuperSonic^®^ Aixplorer.

### Operating Room Protocol

**Figure 1:**
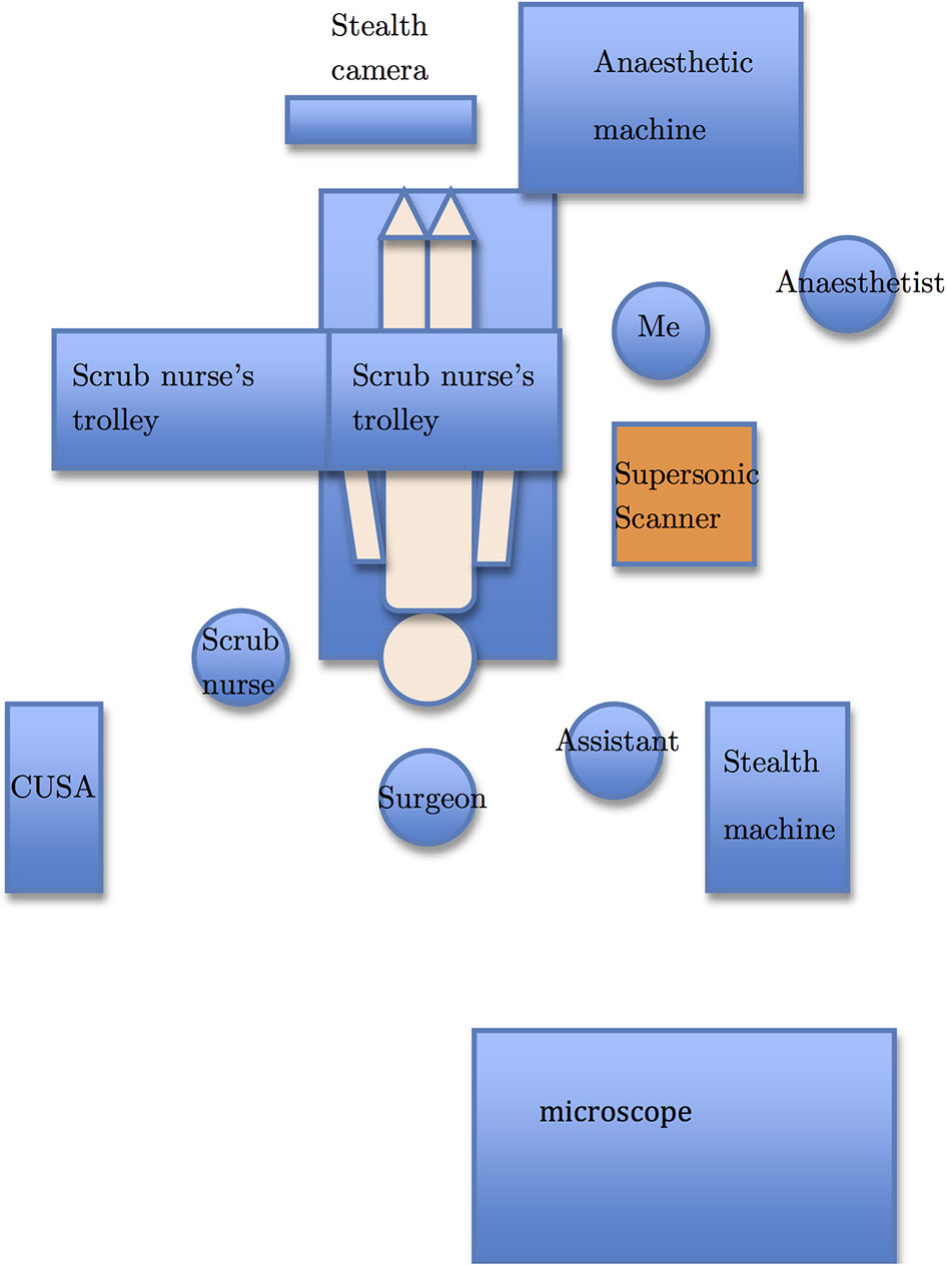
The operating room setup. The Supersonic Aixplorer^®^ scanner (orange) is placed next to the patient body (beige) with the monitor swivelled to face the surgeon. I stand next to the scanner so that I can optimize the scanner settings as well as acquire images for this thesis.

The operating room setup is depicted in **Figure 1**. After anesthesia, the patient was transferred to the operating table and his/her head was pinned with a Mayfield clamp to immobilize the head. Stealth^®^ neuronavigation registration was performed to plan the craniotomy placement. The location of the tumor was determined with neuronavigation. For intrinsic tumors, SWE was performed after durotomy and prior to corticotomy, whereas SWE was performed prior to durotomy for extrinsic tumors as they are often adhere to the dura. After the initial scan, the resection was initiated. The resection was continued until such time when the surgeon felt that he wanted to check the extent of resection or to confirm the location of the tumor. At this time, IOUS was again performed with SWE simultaneously to assess the extent of resection or to confirm the location of the tumor. Final SWE was performed to assess the final extent of resection prior to closure of craniotomy.

### Data Acquisition

After craniotomy, the ultrasound transducer was placed in a sterile sheath filled with acoustic coupling gel. The SWE mode on the SuperSonic Aixplorer^®^ scanner was then activated. The scans were performed on exposed cortex for intrinsic tumors and on closed dura for extrinsic tumors. The intraoperative SWE scans were performed using Aixplorer^®^ (SuperSonic Imagine, France) using a sector transducer (SE12-3) and a linear transducer (SL15-4) with a bandwidth of 3-12 MHz and 4-15 MHz, respectively, using the SWE mode. The sector transducer was used at Great Ormond Street Hospital whereas the linear transducer at the National Hospital for Neurology and Neurosurgery. Without informing the surgeon about the SWE findings beforehand, the surgeon was asked to grade the stiffness of the lesions from 1 to 5 as follows:

1. The lesion is very soft like cyst.
2. The lesion is softer than brain.
3. The lesion is similar to brain.
4. The lesion is stiffer than brain.
5. The lesion is very stiff like cartilage.

After the resection was deemed complete by the surgeon, SWE was performed to determine if there was tumor residual. The tumor residual was graded by the author as either present or absent on both SWE and B-mode. The Q-box function was used to measure the Young’s modulus for the tumor bed and adjacent brain. The YM contrast (YMC) was calculated using the following equation:

$$YMC=\frac{E_{l}-E_{b}}{E_{l}+E_{b}},$$

where *E_l_* is the YM of the lesion and *E_b_* is the YM of adjacent normal brain. A negative YMC denotes a soft tumor whereas a positive YMC a stiff tumor.

### Data Analysis

The intra-operative findings by SWE, B-mode and the surgeon were compared with post-operative MRI.

Statistical analysis using Spearman’s rank correlation, a non-parametric statistical test, was performed for comparison of Young’s modulus measurements and Young’s modulus contrast with surgical findings, because the grading of stiffness was ordinal and discrete whereas Young’s modulus and Young’s modulus contrast were continuous. McNemar’s test was used to compare SWE, B-mode and surgeon’s opinions with post-operative MRI, which was considered the “gold standard”, and also SWE with surgeon’s opinions. This test uses 2 × 2 contingency tables with dichotomous, that is either a “Yes” or “No” in this case, result for paired data. Statistical analysis was performed using Student’s t-test and Mann Whitney U test to compare Young’s modulus measurements for different histological diagnoses, when the Young’s modulus distribution was Gaussian and non-Gaussian, respectively. For paired data comparing Young’s modulus measurements for brain tumors and corresponding surrounding brain, paired Student’s t-test and Wilcoxon signed-rank test were used for normally and non-normally distributed data.

## Results

A total of 34 patients were recruited into the study. The summary of the cases is given in **Table 1**.

**Table 1:** Summary of brain resection cases recruited into this study.

Patient number	Age	Gender	Diagnosis	YM (kPa)(mean ± SD)	YMC	Stiffness grading	Residual (Surgeon)	Residual (B-mode)	Residual (SWE)	Residual (MRI)	US Probe
1	12	M	Low grade astrocytoma	26.2 ± 3.1	0.297	2	No	Yes	No	No	Sector
2	11	F	sPNET^£^	35.6 ± 4.5	0.361	2	No	Yes	Yes	Yes	Sector
3	1	F	Choroid plexus papilloma	13.2 ± 2.6	−0.064	2	No	Not done	Not done	No	Sector
4	6	F	Epidermoid cyst	182.4 ± 15.6	0.772	4	No	No	No	Yes	Sector
5	11	F	Residual sPNET^£^	164.4 ± 48.4	0.692	4	No	No	No	No	Sector
6	15	M	Pilomyxoid astrocytoma	9.7 ± 1.7	−0.224	2	Yes	Yes	Yes	Yes	Sector
7^$^	8	F	SEGA^§§^	300 ± 0	N/A	4	No	No	Yes	No	Sector
8^$^	6	F	SEGA^§§^	300 ± 0	N/A	4	No	No	No signal	No	Sector
9	6	M	Metastasis from clear cell sarcoma of the kidney	241.6 ± 21	0.788	5	No	No	Yes	Yes	Sector
10	15	M	GBM^$$^	154.4 ± 20.9	0.748	4	Yes	Yes	Yes	Yes	Sector
11	2	F	Pilocytic astrocytoma	17.8 ± 1.5	−0.285	2	No	Yes	Yes	Yes	Sector
12	2	M	ATRT^&&^	159.1 ± 82.3	0.802	5	Yes	No	Yes	Yes	Sector
13	14	M	Recurrent pleomorphic xanthoastrocytoma	197.8 ± 2.4	0.681	N/A	Yes	Yes	Yes	Not done*	Sector
14	1	F	ETANTR^££^	4.2 ± 0.9	−0.720	2	Yes	Yes	Yes	Not done^§^	Sector
15	3.3	M	Pineoblastoma	196.3 ± 23.6	0.920	4	Yes	Yes	Yes	Yes	Sector
16	1.1	M	Choroid plexus papilloma	11.9 ± 5.9	−0.290	2	No	No	No	No	Sector
17	7	F	Anaplastic ganglioglioma	11 ± 3.9	−0.102	2	No	No	No	No	Sector
18	17	F	Pleomorphic xanthoastrocytoma with anaplasia	11.2 ± 1.4	−0.138	2	Yes	Yes	Yes	Yes	Sector
19	15	M	Recurrent pilocytic astrocytoma	146.4 ± 14.1	0.889	4	Yes	Yes	Yes	Yes	Sector
20	43	F	Meningioma	39.5 ± 1.2	0.771	4	No	Yes	No	No	Linear
21	1	M	ATRT^&&^	33.6 ± 8.6	0.559	2	No	Yes	No	No	Sector
22	1	F	Residual choroid plexus papilloma	178.9 ± 57.6	0.921	4	No	Yes	Yes	Yes	Sector
23	53	M	GBM^$$^	7.3 ± 3.2	−0.170	2	No	No	Yes	Yes	Linear
24^&^	39	M	Residual medulloblastoma	33.1 ± 8.6	0.458	3	No	N/A	N/A	No	Linear
25	61	F	GBM^$$^	12.3 ± 1.3	0.070	2	No	No	Yes	Yes	Linear
26	46	F	GBM^$$^	3 ± 0.9	−0.439	2	No	Yes	Yes	Yes	Linear
27	62	M	Vestibular schwannoma	153.8 ± 56.3	0.870	4	Yes	Yes	Yes	Yes	Linear
28	49	F	Meningioma	5.6 ± 2	−0.158	2	No	Yes	No signal	Yes	Linear
29	40	M	GBM^$$^	9.9 ± 4.9	−0.823	2	No	Yes	No signal	Yes	Linear
30	35	F	Pilocytic astrocytoma	31.8 ± 5.7	0.216	2	No	Yes	Yes	No	Linear
31	49	F	Metastasis from breast	97.6 ± 42	0.711	4	No	Yes	Yes	Yes	Linear
32	56	F	Meningioma	46.2 ± 24.2	0.505	4	No	No	No	No	Linear
33	5	F	Pilocytic astrocytoma	11.4 ± 0.7	−0.088	2	No	Yes	Yes	Yes	Sector
34	10	M	Anaplastic ependymoma	77.1 ± 11.4	0.858	2	No	No	No signal	No	Sector

^¶^Standard deviation. *It was converted to biopsy due to excessive bleeding. ^§^It was a case of known residual due to invasion into brainstem. ^&^The patient developed air embolus during surgery so the post-operative scan was abandoned. ^$^The calculation of YMC for these cases was not possible as the lesions were subventricular and the adjacent brain was too deep for shear wave to penetrate. ^£^Supratentorial primitive neuroectodermal tumor. ^§§^Subependymal giant cell astrocytoma. ^&&^Atypical teratoid/rhabdoid tumor. ^$$^Glioblastoma multiforme. ^££^Embryonal tumor with abundant neuropil and true rosettes.

The patient characteristics are summarized in **Table 2**.

**Table 2:** Summary to patient and tumor characteristics.

Variables		n
Age		20.7y (range: 1–62y)
Sex	Female	19
	Male	15
Hispathology	High grade glioma	8
	Low grade glioma	9
	Metastasis	2
	Meningioma	3
	Choroid plexus papilloma	3
	Developmental	1
	Vestibular schwannoma	1
	Malignant embryonal tumor	6
Tumor locations	Frontal	11
	Temporal	7
	Parietal	5
	Thalamus	1
	Posterior fossa	9
	Pineal	1

### Comparison of Young’s Modulus Measurements With Surgical Findings

One patient (Patient 13) was excluded for comparison because the surgeons could not ascertain the stiffness of the tumor due excessive bleeding upon opening the dura, which resulted in the operation being abandoned.

**Figure 2:**
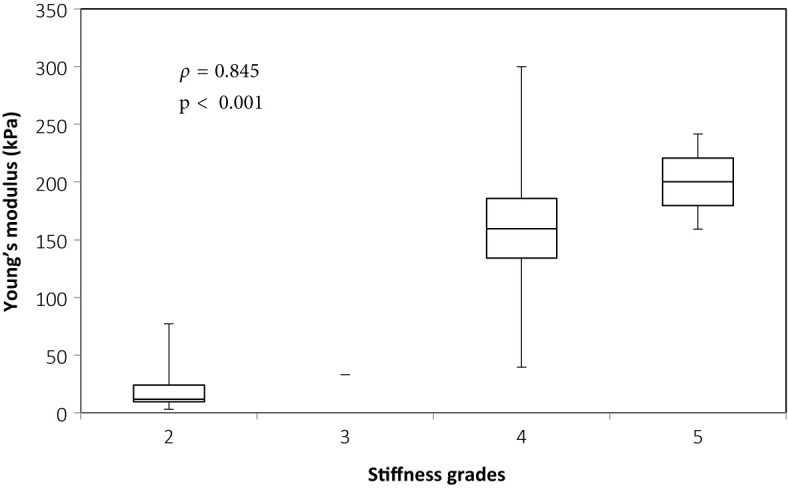
Box and whiskers plot for Young’s modulus measurements according to stiffness grade assessed by independent surgical opinion (n = 34). The upper and lower bounds of the box were the third and first quartiles, respectively, while the line within the box was the median. The upper and lower whiskers were maximum and minimum values, respectively.

**Figure 3:**
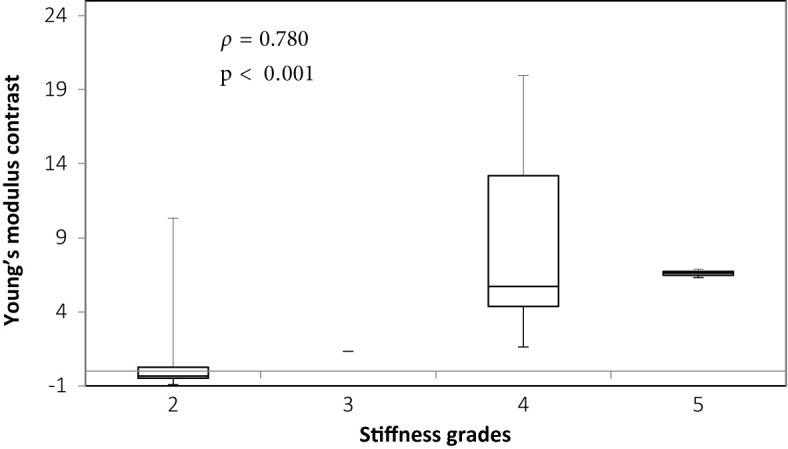
Box and whiskers plot for Young’s modulus contrast according to stiffness grade assessed by independent surgical opinion (n = 32). The upper and lower bounds of the box were the third and first quartiles, respectively, while the line within the box was the median. The upper and lower whiskers were maximum and minimum values, respectively.

**Figure 4:**
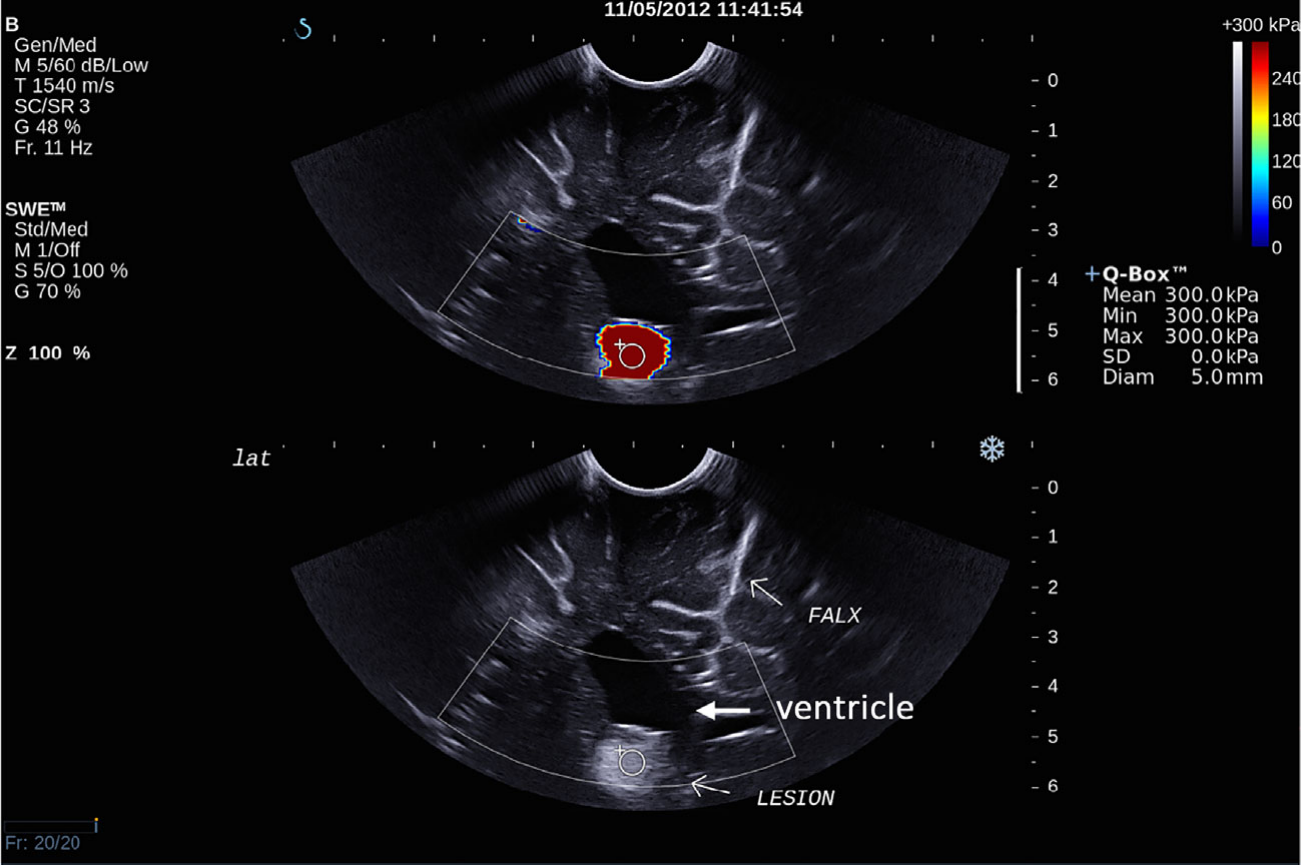
Intra-operative SWE for Patient 7 showing the tumor lying under the ventricles. The adjacent deep grey matter did not have any SWE signal. This scan was acquired using the sector probe (SE12-3) insonating in the coronal plane. The histology of this lesion is subependymal giant cell astrocytoma (SEGA).

**Figure 5:**
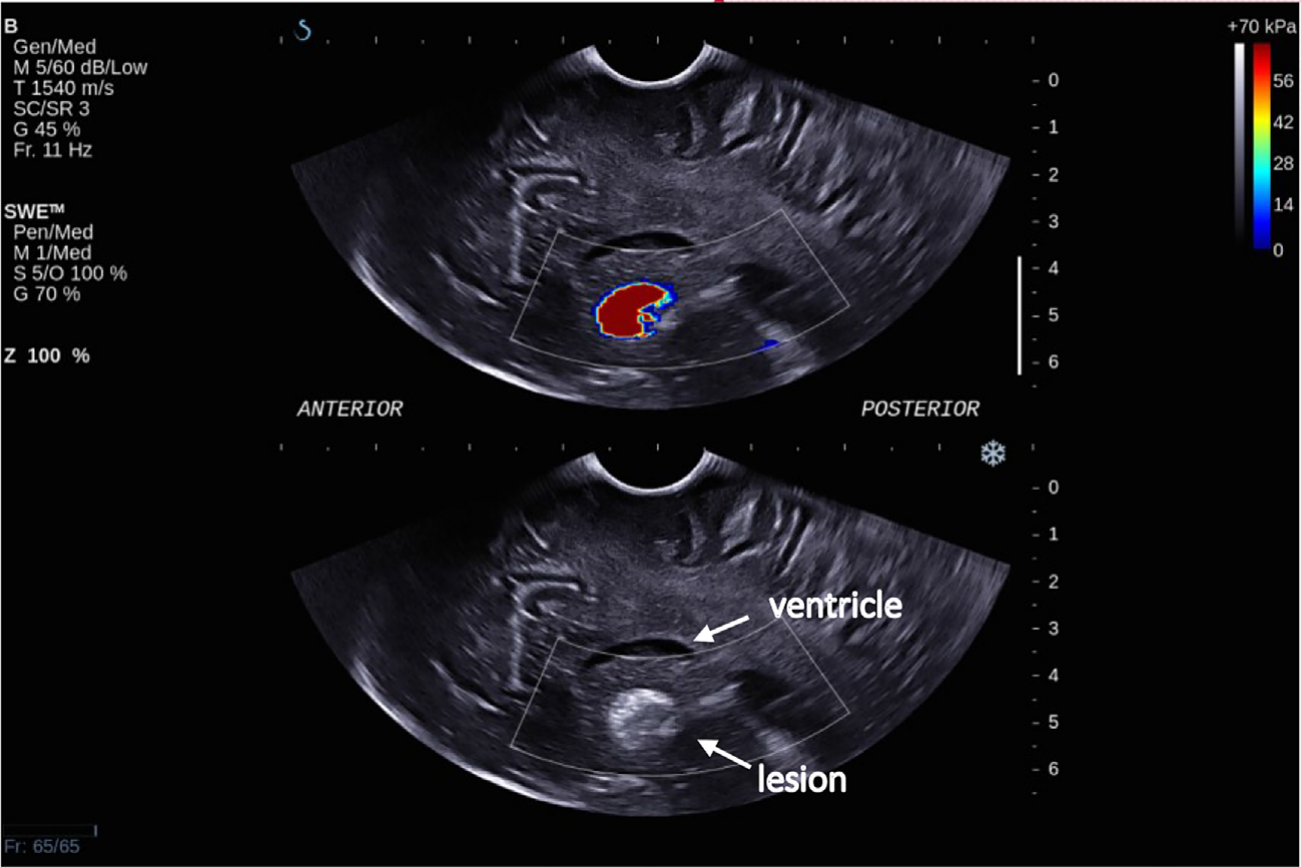
Intra-operative SWE for Patient 8 showing the tumor lying under the ventricles at a depth of 5 cm from the transducer. There was a lack of SWE signal in the adjacent brain. This scan was acquired using the sector probe (SE12-3) insonating in the sagittal plane. The histology of this lesion again is subependymal giant cell astrocytoma (SEGA).

There was overall a significant correlation between Young’s modulus measurements and surgical grading (Spearman’s rank correlation coefficient (*ρ*) = 0.845, p < 0.001), illustrated in **Figure 2**. The correlation between the Young’s modulus contrast and surgical grading was also significant (*ρ* = 0.780, p < 0.001), as illustrated in **Figure 3**. The calculation of Young’s modulus contrast for Patients 7 and 8 was not possible as the tumor, SEGA, was located under the ventricle and the adjacent brain could not be imaged with SWE due to lack of signal (see **Figures 4** and **5**).

**Figure 6:**
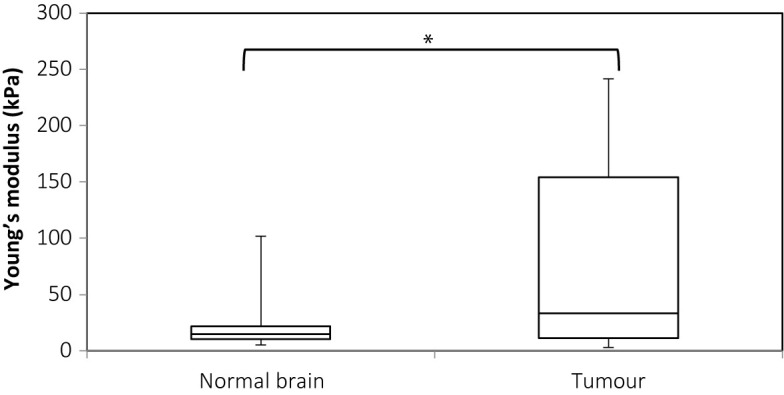
Box and whiskers plot for Young’s modulus measurements of normal brain and tumor. The upper and lower bounds of the box were the third and first quartiles, respectively, while the line within the box was the median. The upper and lower whiskers were maximum and minimum values, respectively. *There was a significant difference between the two groups (p = 0.003, Wilcoxon’s signed-rank test).

**Figure 7:**
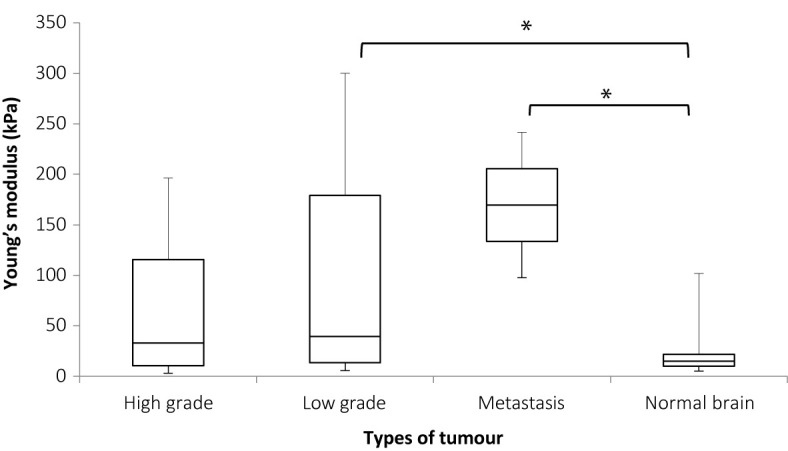
Box and whiskers plot of Young’s modulus distributions for various tumor types. The upper and lower bounds of the box were the third and first quartiles, respectively, while the line within the box was the median. The upper and lower whiskers were maximum and minimum values, respectively. *There was a significant difference between the Young’s modulus values for low grade tumor and normal brain (p = 0.036) and between metastases and normal brain (p = 0.007) but the differences between high grade and normal brain (p = 0.125), high grade and low grade (p = 0.220), metastases and high grade (p = 0.132), and low grade and metastasis (p = 0.288), were not significant. Mann Whitney U test was used to perform statistical analysis.

**Figure 6** shows the plot for Young’s modulus of normal brain and all tumors. The median Young’s modulus for the normal brain was 14.9 kPa, which was significantly lower than that for all tumors (median 33.5 kPa, p = 0.003). Wilcoxon’s signed-rank test was used to perform paired statistical analysis, as the values for both the tumor and normal brain were not normally distributed. **Figure 7** shows the Young’s modulus for various tumor types. The tumors with WHO grades of I and II were graded as low grade while those with WHO grades of III and IV were graded as high grade. As demonstrated, the low grade and high grade tumors were not normally distributed, hence the Mann Whitney U test was used to judge whether the difference between the values in the two groups were statistically significant. Low grade tumors tended to be stiffer than high grade tumors, and metastases even stiffer. However, differences between the Young’s moduli were not significant for low grade versus high grade (p = 0.220), low grade versus metastasis (p = 0.288), and high grade versus metastasis (p = 0.101).

### Residual Tumor Detection

**Table 3** shows the 2 × 2 contingency table comparing SWE with MRI findings on the presence of residual tumor. Using MRI as the “gold standard”, the sensitivity and specificity of SWE for detection of residual tumor identified by MRI were 94% and 77%, respectively. McNemar’s test showed no statistically significant difference between SWE and MRI in the number of cases in which residual tumor was and was not detected (p = 1.000).

**Table 3:** McNemar’s 2 × 2 contingency table comparing intra-operative SWE with post-operative MRI for detecting residual tumor.

MRI	Residual tumor	No residual tumor	Total
SWE			
**Residual tumor**	16	2	**18**
**No residual tumor**	1	7	**8**
**Total**	**17**	**9**	**26**

No statistically significant difference was detected between SWE and MRI for detecting residual tumor (p = 1.000).

The sensitivity and specificity of US B-mode for the same task were 73% and 63%, respectively, using MRI as the “gold standard” (*see* **Table 4**). McNemar’s test showed no statistically significance between US B-mode and MRI for detecting residual tumor (p = 1.000).

**Table 4:** McNemar’s 2 × 2 contingency table for comparing intra-operative US B-mode with post-operative MRI scan in detecting residual tumor.

B-mode	Residual tumor	No residual tumor	Total
MRI			
**Residual tumor**	14	4	**18**
**No residual tumor**	5	7	**12**
**Total**	**19**	**11**	**30**

No statistically significant difference was detected between US B-mode and MRI for detecting residual tumor (p = 1.000).

When comparing surgeon’s findings with MRI, there was a statistically significant difference (p < 0.001), as illustrated in **Table 5**. The sensitivity and specificity of surgeon’s opinion on whether residual tumor was present were 36% and 100%, respectively.

**Table 5:** McNemar’s 2 × 2 contingency table for comparing surgeon’s intra-operative opinion with post-operative MRI scan in detecting residual tumor.

MRI	Residual tumor	No residual tumor	Total
Surgeon			
**Residual tumor**	7	0	**7**
**No residual tumor**	12	13	**25**
**Total**	**19**	**13**	**32**

A statistically significant difference was detected between surgeons’ opinion and MRI for determining whether there was residual tumor or not (p < 0.001).

When comparing SWE with US B-mode, there was no statistically significant difference between them (p = 0.727), as shown in **Table 6**.

**Table 6:** McNemar’s 2 × 2 contingency table for comparing intra-operative SWE with B-mode in detecting residual tumor.

B-mode	Residual tumor	No residual tumor	Total
SWE	Residual tumor	No residual tumor	Total
**Residual tumor**	15	5	**20**
**No residual tumor**	3	5	**8**
**Total**	**18**	**10**	**28**

No statistically significant difference was detected for SWE and B-mode for detecting residual tumor (p = 0.727).

When comparing SWE against surgeon’s findings, there was a statistically significant difference between them (p = 0.001), as shown in **Table 7**. Although surgeon agreed with SWE when SWE demonstrated no residual tumor, SWE detected residual tumor in significantly more cases than surgeon.

**Table 7:** McNemar’s 2 × 2 contingency table for comparing intra-operative SWE and surgeon’s opinion in detecting residual tumor.

Surgeon	Residual tumor	No residual tumor	Total
SWE	Residual tumor	No residual tumor	Total
**Residual tumor**	9	11	**20**
**No residual tumor**	0	8	**8**
**Total**	**9**	**19**	**28**

A statistically significant difference was deteted for SWE and surgeon’s opinion of the presence of residual tumor. (p = 0.001).

Of the 34 patients, only 26 patients were included in the comparison between intra-operative SWE and post-operative MRI in detecting residual tumor. Patients 13 and 14 were excluded because post-operative MRI was not performed. Patient 13 developed excessive bleeding during surgery resulting in the surgeons abandoning the operation. Patient 14 had extensive invasion into the brainstem on pre-operative MRI. Therefore, the surgeons decided to perform only debulking surgery with intended residual tumor. Patients 8, 11, 28, 29, and 34 did not have SWE signal post-operatively despite using “penetration mode” and the probe being held as close to the cavity as possible. The tumor in Patient 8 was situated at a depth of >5 cm, and there was no SWE signal. In contrast, Patient 7, similar case to Patient 8, had SWE signal down to a depth of 5-cm post-resection, but the signal was of questionable reliability as it gave a very high Young’s modulus of 300 kPa. For Patient 34, the water standoff could not be maintained in the resection cavity. For cases performed at the National Hospital for Neurology and Neurosurgery, as the acquisition was performed using a linear array probe, which was a lot larger than the sector array probe, all the post-resection scans were performed with the probe above the cavity using a water standoff. Patient 28 had Surgicel^®^ in the resection cavity prior to the scan because of excessive bleeding, thereby causing a lack of SWE signal. Patient 29 had a large GBM prior to resection, resulting in a very deep post-resection cavity. Due to the large linear array probe, it was not possible to insert the probe in the cavity. Patient 3 did not have a post-resection scan, as it was one of the earlier cases where the primary aim was to investigate the feasibility and determine the artifacts associated with clinical scanning. Patient 24 did not have a post-resection scan as the patient developed an air embolus and the surgery had to be abandoned.

## Discussion

Neurosurgeons usually employ intra-operative tools, which offer spatial orientation, navigational guidance, and up-to-date imaging to achieve maximal brain tumor resection. However, as these intra-operative tools do not offer elasticity imaging, to gain information on tissue mechanics neurosurgeons ultimately rely on visual inspection and tactile feedback during surgery. Neurosurgeons tend to overestimate the extent of resection of brain tumors by up to three times, as judged by post-operative MRI (34, 35). This could be due to the similarity in appearance of tumor and brain resulting in the neurosurgeon having difficulty in differentiating tumor from brain.

Elastography is a term coined in 1991 to describe a method of quasistatic ultrasound strain imaging (37). Nowadays, there are three main types of ultrasound elastography, namely: quasistatic strain elastography (QSE) (37), shear wave elastography (SWE) (38–42), and acoustic radiation force impulse (ARFI) imaging (44). Ultrasound elastography has been employed in clinical practice to characterize lesions in other parts of the body including the salivary gland, the thyroid, the breast, the gastrointestinal tract, the prostate, and the liver (45). Although ultrasound elastography in the brain has not been used in clinical practice, there have been studies looking at the application in brain tumor surgery using QSE (46–51) and SWE (52, 53). Chakraborty et al. (47) demonstrated feasibility of using QSE co-registered with MRI in brain tumor resection whereas Uff et al. (46) showed that real-time QSE was able to demonstrate good correlation between surgeon and elastograms in determining tumor stiffness and surgical plane. Selbekk et al. (50) and Selbekk, Bang, and Unsgaard (51) demonstrated that arterial pulsations were able to generate elastograms to improve visualization of tumors. Cepeda et al. (49) showed that peritumoural tissue of glial tumors have different elasticity compared to other tumor types. Prada et al. (48) showed that elastograms were superimposable to US B-mode and had sharper tumor margins than US B-mode. Besides brain tumor surgery, ultrasound elastography has also been studied in paediatric (54, 55) and epilepsy surgery (56). Su et al. (54) showed that the brains in preterm babies have lower stiffness compared to those in term babies whereas Kim et al. (55) demonstrated different stiffness in different parts of neonatal brains. Chan et al. (56) demonstrated a case report of the detection of MRI-negative epileptogenic lesion using SWE. There have also been studies using ultrasound elastography in animal models looking at changes in brain elasticity after stroke (57) and trauma brain injury (58).

### Comparison of Young’s Modulus Measurements With Surgical Findings

This study showed that there was significant correlation between both Young’s modulus and Young’s modulus contrast measured by SWE and surgical opinion of tumor stiffness relative to normal brain. In the brain tumors, the Young’s modulus measurements agreed better than Young’s modulus contrast with surgical grading of the stiffness. This is consistent with the findings in other studies using QSE where strain was correlated with surgical opinion on tumor stiffness (47, 53, 59, 60). In Patient 34, although the tumor was thought to be softer than brain by the surgeon, the Young’s modulus measurement was 77.1 ± 11.4 kPa (mean ± SD) and Young’s modulus contrast was 0.858, indicating that it was stiffer than brain by SWE. This could be due to undue pressure on the brain surface when performing the scan. The location of the tumor, which was parietal, was particularly vulnerable to saline irrigation flowing out making water standoff almost impossible. Therefore, in order to acquire a good quality B-mode or SWE, the surgeon might have applied too much pressure, which was known to cause artifactual stiffness due to the non-linear effects of pre-compression in the area of stress concentration caused by the transducer. As the lesion was superficial, this effect could be unintentionally produced by small pressure.

This study showed that the Young’s modulus for all tumor types was significantly higher than normal brain (p = 0.003), thereby showing that SWE was capable of differentiating various brain tumors from the surrounding brain. The paired statistical analysis, Wilcoxon’s signed rank test in this case, showed that the difference in Young’s modulus measurements was significant between brain tumors and normal brain. In this study low grade tumors tended to be stiffer than high grade tumors, and metastases stiffer still, although the differences between median values was not significant. However, the low grade tumors (p = 0.036) and metastasis (p = 0.007) were shown to be significantly stiffer than brain. Murphy et al. (61) showed that meningiomas have shear modulus of 2–10 kPa, that is, Young’s modulus of 6–30 kPa. From the intraoperative SWE study by Chauvet et al. (62), the meningiomas was found to have a Young’s modulus of 33.1 ± 5.9 kPa. From **Table 1**, the meningioma cases (patients 13, 30, and 31) showed that the Young’s modulus ranged from 5.6 to 46.2 kPa (mean 30.4 kPa; SD 21.8 kPa), which agreed with the literature. In a case of breast metastasis (NHNN 14), the Young’s modulus was 97.6 kPa ± 42 kPa (mean ± SD), which agrees with the reported values of 61–165 kPa in the literature (63, 64). This study attempts to examine whether Young’s modulus is characteristic of brain tumor type, showing that there is considerable variability within groups but due to small numbers of different tumor types, the result was not conclusive. This means that SWE was capable in distinguishing brain tumors from normal brain but was unable to separate different tumor types from each other intraoperatively prior to resection. Chauvet et al. (62) showed that low grade gliomas, meningiomas, and metastasis were significantly stiffer than normal brain. The high grade glioma is stiffer than normal brain but the result was not statistically significant. In this study the normal brain only had Young’s modulus of 6.3 to 7.2 kPa, which was much lower than the findings from this thesis (median Young’s modulus of 31.0 kPa and 18.8 kPa for grey and white matter, respectively). This study also showed that the different tumor types had significantly different Young’s modulus, which was not shown by this study. This could be due to the much larger number of patients in this study (63 patients) than this study (34 patients).

### Residual Tumor Detection

Using post-operative MRI as the “gold standard”, detection of residual tumor by SWE was shown to have a sensitivity and specificity of 94% and 77%, respectively. Currently, there has been no literature reporting the sensitivity and specificity of SWE in detecting residual brain tumor. SWE was shown to be comparable to post-operative MRI in detecting residual tumor.

Compared to post-operative MRI, US B-mode was shown to have a sensitivity and specificity of 73% and 63%, respectively, for detecting residual tumor. This result agrees with current literature results, which showed a sensitivity of 67%–85% (26, 27, 30). US B-mode was also shown to be comparable to both post-operative MRI findings and SWE in detecting residual tumor.

Surgeon’s intraoperative opinion of the presence or absence of residual tumor was shown to be significantly different from post-operative MRI findings. The sensitivity of surgeon’s opinion in detecting residual tumor was also lower than both SWE and US B-mode. This agrees with Albert et al. (34) and Orringer et al. (35), where surgeons were less likely to detect residual tumor than post-operative MRI. The reason underpinning this could be that tumor can have similar appearance to normal surrounding brain, making visual inspection less reliable. Furthermore, after manipulation and dissection, the tumor typically becomes softer and loses its pre-resection appearance, thereby making it harder for the surgeon to differentiate it from normal surrounding brain. Most surgeons would err on the side of caution to prevent neurological deficit, thereby explaining the lower sensitivity of surgeon detecting residual tumor. Having said that, surgeons had a specificity of 100%, higher than both SWE and US B-mode, in detecting residual tumor. This means that when there was no residual tumor on the MRI scan, surgeon would correctly identify this intraoperatively. Comparing SWE with surgeon’s findings, SWE significantly detected more cases of residual tumor than surgeon. This could be explained by the ability of SWE to visualize the tumor deep to the “manipulated” tumor. Therefore, using SWE in combination with surgeon’s opinion may optimize the detection of residual tumor as SWE has a higher sensitivity than surgeon while the surgeon has a higher specificity than SWE.

## Limitations

This study has a few limitations. Firstly, the small sample size with a heterogeneous tumor types in this study, has led to statistically non-significant results in the ability for SWE to differentiate different tumor types. This can be overcome with a larger study focusing on main tumor types undergoing resection. Secondly, the use of two different of US probes in the two different hospitals in which the study was performed, may pose some skepticism regarding the validity of the SWE measurements. However, the measurements of the normal brain Young’s modulus were shown to be similar using these two different probes. Furthermore, the bandwidths for these probes were very similar—3-12 MHz and 4-15 MHz for the sector and the linear probes, respectively, thereby producing very similar image quality. Thirdly, due to SWE signal insufficiency, there were eight patients (24%) excluded from SWE analysis compared to four patients (12%) and two patients (6%) excluded from B-mode and surgeons’ opinion analyses. This may have biased the result, which again can be overcome with a larger sample.

## Conclusion

This study showed that the SWE measurements of Young’s modulus and Young’s modulus contrast correlated significantly with surgical grading of stiffness. This means that clinically, the SWE measurements are reliable in predicting stiffness.

It also showed that there was high sensitivity and specificity of SWE in detecting residual tumor compared to post-operative MRI scan as the “gold standard”. It also showed that when there is residual tumor, SWE is better than the surgeon at detecting residual tumor by 2.5 times (94% versus 37%). When there is an absence of residual tumor, the surgeon is better at predicting the absence of residual tumor (100% versus 77%). These results imply that intraoperative SWE can be a useful tool in assisting neurosurgeons in identifying residual tumor during resection. However, these results are preliminary due to the small sample size with heterogeneous tumors. A larger study with less heterogeneous tumor types will be required to show reproducibility of these findings.

## Data Availability Statement

The raw data supporting the conclusions of this article will be made available by the authors, without undue reservation.

## Ethics Statement

The studies involving human participants were reviewed and approved by National Research Ethics Service (NRES) Committee London—Queen Square (REC reference: 08/ H0716/92; amendment number: AM04). Written consents were provided by the participants, or the participants’ legal guardian/next of kin if the participants were unable to provide consents.

## Author Contributions

HC conceived and designed the analysis, collected the data, performed the analysis, and wrote the paper. CU, AC, ND, and JB contributed data and analysis tools, and performed the analysis. All authors contributed to the article and approved the submitted version.

## Funding

The authors would like to thank the Royal Free Charity and Royal Free Clinical Research Fellowship for funding the study. The authors would also like to thank the Engineering and Physical Sciences Research Council (EPSRC) for funding the equipment.

## Conflict of Interest

The authors declare that the research was conducted in the absence of any commercial or financial relationships that could be construed as a potential conflict of interest.
